# Resident memory macrophages and trained innate immunity at barrier tissues

**DOI:** 10.7554/eLife.106549

**Published:** 2025-10-20

**Authors:** Alisha Kang, Michael D'Agostino, Sam Afkhami, Mangalakumari Jeyanathan, Zhou Xing

**Affiliations:** 1 McMaster Immunology Research Centre, Department of Medicine, M.G. DeGroote Institute for Infectious Disease Research, McMaster University Hamilton Canada; Queen's University Canada; National Institute of Immunology India

**Keywords:** trained immunity, memory macrophages, barrier tissues, lung, vaccine

## Abstract

Innate immune memory, or trained innate immunity (TII), represents a form of immunological adaptation in which innate immune cells, including myeloid and lymphoid cells, retain a trained state following prior exposure to immunological stimuli. This long-lasting modification either enhances or reduces the innate immune response to subsequent heterologous infections or inflammatory insults. While TII often provides protective benefits, including enhanced protection against pathogens and tumors, it can contribute to maladaptive inflammation in certain conditions. Epigenetic changes and metabolic reprogramming are key drivers of innate immune memory, but it is important to distinguish between transient acute changes and persistent modifications that define bona fide innate immune memory. Innate immune memory can be induced centrally, through systemic events that train hematopoietic progenitors in the bone marrow, or locally, via tissue-resident cells such as macrophages. The presence of trained tissue-resident immune cells offers significant advantages, but their responses may not always result in universally enhanced protection. This review explores recent advances in the understanding of tissue-resident memory macrophages and TII at barrier tissue sites, including the lung, skin, gut, and peritoneum, highlighting the implications for vaccine and immunotherapeutic strategies. Ongoing research promises to accelerate progress in this field and inform new clinical and vaccinology approaches.

## Introduction

Over the past decade or so, there has been a significant expansion in our knowledge of innate immune memory and trained innate immunity (TII) (Netea et al., 2016). Although the phrases innate immune memory and TII are often interchangeably used in literature, it is our view that to avoid confusion and given its functional connotation, we should reserve the use of TII only for when we refer to innate immune protective outcomes following induction of innate immune memory. Innate immune memory represents the adaptive state of innate immune cells, including myeloid and lymphoid innate cells, as well as structural barrier cells, that persists long after previous immunological exposure (Domínguez-Andrés et al., 2023; Xing et al., 2020). Such imprinted or trained innate cells lead to an altered, either strengthened or weakened, innate immune response upon subsequent homologous or heterologous immunological exposure. While the strengthened innate immune response often functionally offers enhanced innate immune protection or TII against pathogens or tumor cells (Domínguez-Andrés et al., 2023), it may underpin the maladaptive consequences of inflammatory conditions (Halper-Stromberg and Jabri, 2022). Innate immune memory is sometimes referred to as inflammatory memory (Ordovas-Montanes et al., 2020), and the process of its development is considered to be ‘inflammatory adaptation’ (Niec et al., 2021). Epigenetic modifications and metabolic rewiring are involved in the development and maintenance of innate immune memory (Ferreira et al., 2024; Sun and Barreiro, 2020). However, the short-term activation or temporary epigenetic modifications should not be confused with the persisting epigenetic changes characteristic of bona fide innate immune memory (Sun and Barreiro, 2020). Thus, the time from the acute immunological episode and whether the initial inflammation or infection has resolved are among important considerations for investigating innate immune memory and TII (Niec et al., 2021).

**Figure 1.**
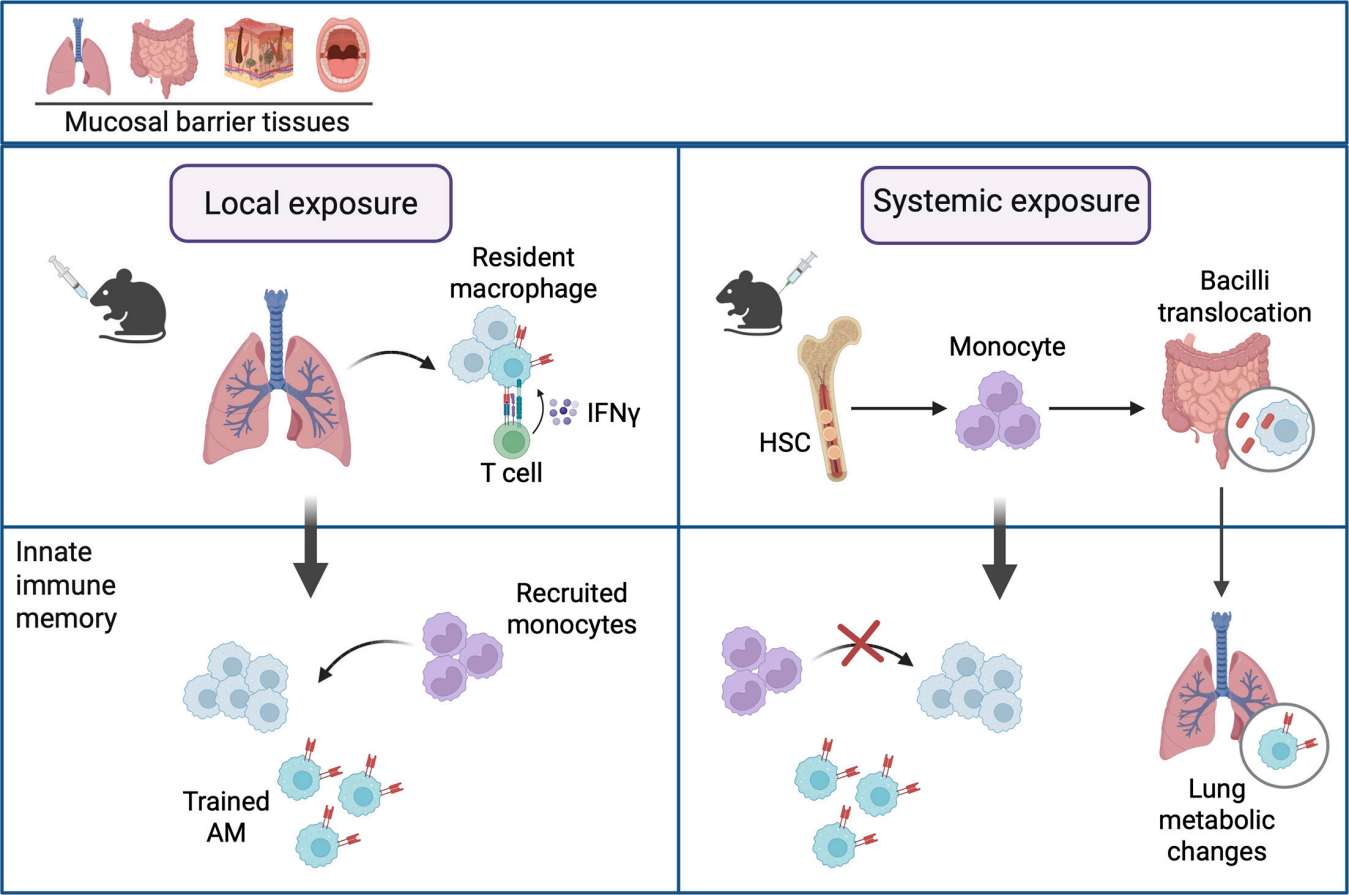
Induction of innate immune memory in tissue-resident macrophages following local and systemic immunological exposure. Following local immunological exposure to immune stimuli, including adenovirus-vectored vaccine, the priming, but not the maintenance, of memory alveolar macrophages (AMs) requires help from effector CD8^+^ T cells via IFN-γ production and is contact-dependent. Tissue-resident macrophages at barrier sites trained by vaccines or microbial components causing limited inflammation are primarily of tissue-residential/embryonic origin. Those resulted from more potent stimulation, such as robust infection or severe inflammation, are monocyte-derived AMs. Thus, the degree of inflammation following local exposure can lead to various outcomes, from no replacement to partial or complete replacement of lung tissue-resident macrophages by recruited circulating monocytes. On the other hand, following systemic immunological exposure, such as cutaneous *Bacillus* Calmette-Guérin (BCG) vaccination, BCG bacilli disseminate to gut-associated tissues and lead to a time-dependent changes in the gut microbiota and barrier permeability, and metabolomic changes in the gut, serum, and lung. This results in the induction of memory AMs and TII in the lung. Monocytes play a relatively minor role in the development and maintenance of innate immune memory in lung tissue-resident macrophages, particularly AMs following systemic immunological exposure. Created in https://BioRender.com/478t8uu.

In general, innate immune memory and associated TII can be either centrally or locally induced (Ferreira et al., 2024; Netea and Joosten, 2018; Figure 1). The former often occurs following a systemic immunological event and the training of hematopoietic progenitor cells in the bone marrow and subsequent egress of trained mature myeloid cells such as monocytes and neutrophils into the bloodstream. Such trained circulating myeloid cells may be recruited into the tissue sites in response to locally produced chemotactic signals, thus contributing to TII (Domínguez-Andrés et al., 2023; Kaufmann et al., 2022; Kaufmann et al., 2018). On the other hand, the innate immune memory can arise locally in tissue-resident innate immune cell populations, including macrophages, resulting from a local immunological event (Afkhami et al., 2022; Yao et al., 2018). Recent evidence suggests that tissue-resident memory macrophages could be induced and maintained independently of the recruited blood-derived monocytes (Kang et al., 2024a; Yao et al., 2018). In instances where tissue-resident macrophage populations are largely depleted following an acute episode of infection, the recruited monocytes may adopt a trained phenotype, eventually differentiating to be the tissue-resident memory macrophages but still harboring monocytic gene signatures (Aegerter et al., 2022; Guilliams and Svedberg, 2021). Furthermore, the latest data indicates that locally induced tissue-resident innate immune memory, memory macrophages, or TII could arise in response to a systemic immunological alert or distally derived immunological signals, including gut microbial metabolites (Hoyer et al., 2019; Jeyanathan et al., 2022b; Kang et al., 2024b; Li et al., 2022b; Ngo et al., 2024; Simats et al., 2024). Recent research has demonstrated the remarkable capacity of various tissue sites to accommodate locally elicited tissue-resident innate and adaptive immune memory cells even following successive immunological exposures (Wijeyesinghe et al., 2021). It is of importance to keep in mind that tissue-resident TII may not always be associated with universally enhanced innate immune protection against heterologous pathogens. Mounting evidence suggests that depending on the nature of the heterologous pathogen, there are three likely innate immune outcomes: reduced microbial counts/infection and tissue immunopathology, restrained tissue immunopathology without significant changes in microbial counts/infection, and limited protection in both infection and tissue immunopathology. The second outcome is often seen following acute heterologous respiratory viral infection in the lung and is attributed to enhanced disease tolerance (King and Divangahi, 2019; McCarville and Ayres, 2018). Disease tolerance enhanced through TII or mediated by other mechanisms leads to improved survival of the host via limiting tissue inflammation and injury without altering the magnitude of infection (Afkhami et al., 2022; Damjanovic et al., 2012; Nahrendorf et al., 2021; Pernet et al., 2019). One cannot confuse disease tolerance with innate immune tolerance or weakened innate immune responses rendered by a different type of innate immune memory, upon subsequent homologous or heterologous immunological exposure. Innate immune tolerance often leads to not only heightened magnitude of infection but also excessive tissue immunopathology and injury. For instance, the hosts that have just recovered from acute influenza are especially susceptible to unrestrained bacterial superinfection and immunopathology in the lung (Damjanovic et al., 2013; Small et al., 2010). A firm grasp of differential innate immune protective outcomes by tissue-resident TII is critical to understanding the mechanisms of protection and developing tailored vaccine and immunotherapeutic strategies.

In the present review, we will focus on the progress being made over the past decade in resident memory macrophages and associated TII at selected barrier tissues, including the lung, skin, gut, and peritoneal cavity. Additionally, a brief section is provided in the end to discuss the evidence demonstrating nonimmune epithelial cell-associated TII at barrier tissues. Like other emerging immunological fields, the ongoing progress raises many more questions. We have confidence that the next decade will witness much more accelerated progress and usher in new vaccine and immunotherapeutic strategies into the clinical pipelines.

## Induction of innate immune memory in lung tissue-resident macrophages following local immunological exposure

Innate immune memory may arise, following local immunological exposure, in tissue-resident macrophages within mucosal tissues, specifically the lung (Afkhami et al., 2022; Kang et al., 2024a; Yao et al., 2018). Indeed, recent studies have shown that local immunological exposure can induce innate immune memory in lung tissue-resident macrophages, a phenomenon known as local training, characterized by heightened MHCII expression, increased production of the proinflammatory cytokines IL-6, TNF, and IL-1β, enhanced phagocytic capacity, increased glycolysis, and protection against heterologous infections (D’Agostino et al., 2020; Kang et al., 2024a; Yao et al., 2018; Figure 1). Given its direct external connection, the lungs are particularly conducive to innate immune modulation by airborne microbes and particulate agents. Thus, the induction and the underlying mechanisms mediating innate immune memory in the respiratory mucosa following local immunological exposure remain a priority of investigation. Within the lung, there are distinct subsets of tissue-resident macrophage populations, including alveolar macrophages (AMs), interstitial macrophages (IMs), and monocyte-derived macrophages (MDMs) (Guilliams and Svedberg, 2021). These subsets differ in their ontogeny, with IMs being mainly yolk sac-derived with slow and continuous input from bone marrow-derived monocytes after birth; whereas, on the contrary, AMs are embryonically derived and self-maintain throughout life (Guilliams and Svedberg, 2021). As the most abundant and important innate immune cells in the lung, AMs are positioned at the airway-environment interface and are continuously exposed to airborne microbes and microbial components, which can imprint the local tissue-resident macrophage niche (Martin et al., 2021). Therefore, their immune phenotype and function is subject to regulation by such environmental exposures (Martin et al., 2021).

Recent studies have demonstrated the induction of tissue-resident memory macrophages following respiratory mucosal exposure to microbial components, vaccines, and infections, and type 2 immunity (Th2), and these macrophages are associated with TII. Respiratory mucosal exposure in mice to microbial components, including lipopolysaccharide (LPS), a Gram-negative bacterial cell wall component, and β-glucan, a fungal cell wall component, has been shown to impact local innate immune responses in the lung and modulate AM function through the induction of innate immune memory and TII (Chakraborty et al., 2023; Kang et al., 2024a; Theobald et al., 2024; Zahalka et al., 2022). LPS-mediated type 1 IFN signaling and metabolic rewiring in AMs play a critical role in the induction of innate immune memory (Zahalka et al., 2022). Notably, while glycolysis appears to be dispensable for the establishment of LPS-induced memory AMs, fatty acid oxidation and glutaminolysis are crucial for its development (Kang et al., 2024a; Zahalka et al., 2022). These cells demonstrate plasticity in surface marker expression, acquiring CD11b and downregulating Siglec F, which returns to baseline upon resolution of LPS-induced acute inflammation (Kang et al., 2024a; Chakraborty et al., 2023). At steady state, these memory AMs are maintained independently of monocyte recruitment and proliferation, akin to naïve AMs (Chakraborty et al., 2023; Kang et al., 2024a; Zahalka et al., 2022). Following subsequent infection or tissue injury, memory AMs remain intact and are sustained without a significant contribution from recruited circulating CCR2^+^ inflammatory monocytes. Additionally, LPS-trained AMs exhibit enhanced efferocytosis, a pro-resolving phenotype, via upregulation of MERTK, a key efferocytosis gatekeeper (Chakraborty et al., 2023). MERTK-expressing AMs also show increased chromatin accessibility to the KLA4 transcription factor, which promotes the resolution of pulmonary fibrosis (Chakraborty et al., 2023). This indicates that LPS-trained AMs exhibit not only a proinflammatory phenotype but also a pro-resolving one, helping to prevent hyperinflammation and restore tissue homeostasis (Chakraborty et al., 2023).

A single low-dose intranasal exposure to β-glucan has been shown to induce low-grade inflammation in the lung, resulting in the development of apolipoprotein E-dependent (ApoE) monocyte-derived AMs that exhibit phenotypic and functional changes characteristic of innate immune memory, such as elevated glycolysis and enhanced phagocytic capacity (Theobald et al., 2024). Local respiratory mucosal β-glucan inoculation recruited ApoE^+^ CD11b^+^ circulating monocytes, which were dependent on CCR2 and M-CSF for maintenance. β-Glucan-trained macrophages exhibited phenotypic and functional adaptations capable of improving the outcomes of pulmonary bacterial infection or fibrosis.

Several live-attenuated human vaccines induce innate immune memory in tissue-resident macrophages and exhibit nonspecific protective effects against heterologous respiratory infections (Sánchez-Ramón et al., 2018). The *Bacillus* Calmette-Guérin (BCG) vaccine, the only licensed vaccine for tuberculosis (TB), has been shown to modulate the innate arm of the immune system and induce TII in both experimental models and humans (Netea et al., 2016). Although most of the BCG-induced innate immune memory in tissue-resident macrophages has been described following systemic immunological exposure, experimental respiratory mucosal BCG vaccination has also been shown to induce memory macrophages and TII, which are capable of providing protection against heterologous bacterial infection (Mata et al., 2021). Macrophage activation was dependent on IFN-γ produced by CD4^+^ T cells, suggesting that the induction of trained AMs following respiratory mucosal BCG vaccination requires adaptive immunity (Mata et al., 2021). An important effort was made in this study to differentiate BCG bacillus-mediated macrophage activation from the induction of bona fide memory-like macrophages following BCG vaccination. The claim of a memory macrophage phenotype was not made until BCG bacilli were no longer detectable in the lung using the traditional colony-forming unit assay at 7 months post-BCG vaccination, at which time the cellular responses and inflammatory markers in the lung had largely subsided. Some other TII studies, such as the one by Khan et al., 2025, are limited to immunological analysis performed only during the acute phase and not in the resolution phase of immune responses.

Adenovirus (Ad)-vectored vaccination in murine models has also been shown to induce local innate immune memory in tissue-resident macrophages in the lung and TII against homologous or heterologous respiratory bacterial and viral infections (Afkhami et al., 2022; D’Agostino et al., 2020; Yao et al., 2018). Interestingly, memory AM priming, but not the maintenance, was dependent on the help from adaptive T cells, requiring IFN-γ production by CD8^+^ T cells and cell-cell contact (Yao et al., 2018). The development and maintenance of such memory AMs was independent of circulating CCR2^+^ monocytes. Functionally, Ad-vectored vaccine-induced trained AMs and TII provided markedly enhanced protection against pulmonary *Streptococcus pneumoniae* (*S. pneumoniae*) (Yao et al., 2018) and SARS-CoV-2 infection (Afkhami et al., 2022). For the latter, TII operated via inducing viral disease tolerance but not by altering viral clearance. This contrasts with the training phenotype induced by a single low-dose respiratory LPS exposure, which generated trained AMs and protected against *S. pneumoniae* but not SARS-CoV-2 infection (Kang et al., 2024a). These observations highlight the complexity and differential outcomes of lung-resident memory macrophages and TII, depending on the nature of training stimuli.

Furthermore, the intranasal polymicrobial MV130 vaccine induced airway memory macrophages and TII against heterologous respiratory infections in mice (Brandi et al., 2022; Del Fresno et al., 2021). The composition of the resulting memory airway macrophage population was also independent of circulating monocytes. The development of memory macrophages in the lung was dependent on the mTOR pathway (Brandi et al., 2022). However, one caveat in this study is that repetitive intranasal doses of MV130 may have induced a sustained immunostimulatory/activating effect on tissue-resident macrophages. Nonetheless, the above findings together support the immunological benefits of respiratory mucosal-delivered vaccines in inducing both tissue-resident memory innate and adaptive immune cell types for clinical application. Indeed, the persisting changes have been observed in human AMs following inhaled aerosol Ad-vectored vaccination against TB (Jeyanathan et al., 2022a) or COVID-19 (Jeyanathan et al., 2025) or ex vivo exposure to IFN-γ (Cox et al., 2024).

In addition to attenuated vaccines and microbial components, common respiratory pathogens, including influenza A virus (IAV), SARS-CoV-2, and *S. pneumoniae* that typically cause acute infections, are able to induce airway memory macrophages and TII in experimental models (Aegerter et al., 2020; Guillon et al., 2020; Lercher et al., 2024; Wang et al., 2023). Such memory AMs were of monocytic origin, and their generation was dependent on NK cell-derived IFN-γ. Recruitment of circulating monocytes to the lung following acute IAV infection replenished the depleted AM pool and acquired the phenotype of tissue-resident AMs over time (Aegerter et al., 2020). Recovery from past SARS-CoV-2 infection induced innate immune memory AMs via functional changes associated with innate immune memory and TII against secondary IAV infection (Lercher et al., 2024). Following mild SARS-CoV-2 infection, the imprinted airway-resident macrophage population was maintained independently of circulating monocytes, as there was limited contribution of MDMs to the resident AM pool. Recovered imprinted airway-resident macrophages were enriched in genes associated with antiviral immunity and IFN responses, and type 1 IFN signaling was involved in the development of memory AMs (Lercher et al., 2024). This demonstrates how a history of viral infection can have a significant effect on the subsequent development of disease. Similarly, recovery from pneumococcal pneumonia reprograms the AM pool, and such experienced AMs exhibit characteristics associated with innate immune memory and TII (Arafa et al., 2022). Induction of memory AMs was dependent at least in part on IFN-γ, which was produced by a variety of cells, including CD4^+^ T cells. Following pneumonia, the resident embryonic-derived AM population was replaced by MDMs, which displayed similar phenotypic characteristics as resident AMs. Thus, environmental signals and tissue niche, rather than ontogeny, dictate macrophage phenotype. These findings suggest that exposure to different common respiratory viral and bacterial pathogens can alter the local innate immune landscape and makeup of the tissue-resident macrophage population in the lung depending on the pathogen, severity, and duration of infection.

 While respiratory pathogens have been well characterized to induce innate immune memory and TII in tissue-resident macrophages, noninfectious inflammatory diseases, such as chronic inflammatory diseases and cancer, can also lead to alterations in innate immune cells in the lung (Niec et al., 2021). While the induction and mechanisms of innate immune memory are relatively well understood following local exposure to microbial components, vaccines, and infections, the induction, mechanistic underpinnings, and consequences of innate immune memory and TII induced in Th2 conditions, including allergy and asthma, are only beginning to emerge (Hartung and Esser-von Bieren, 2022). The emerging evidence suggests that house dust mite (HDM)-allergic mice and patients develop M2-like inflammatory memory macrophages in the lung and bone marrow (Lechner et al., 2022). However, the longevity of such HDM-trained lung macrophages and whether they may translate to enhanced innate immune protection remains to be investigated. It is unclear which type of lung-resident macrophages and TII may result from sequential respiratory exposure to an infectious agent/vaccine and an environmental-borne agent such as HDM in the same host. Repeated respiratory immunological exposure is a clinically relevant setting in human lungs where the preexisting TII is expected to be impacted by the effect of a secondary TII-inducing agent and vice versa, likely settling in a re-established and complex innate immune memory environment.

Clearly, depending on the nature of the respiratory mucosal stimuli, the composition of the resulting airway memory macrophage population may differ. While AMs trained by vaccines or microbial components causing limited inflammation are primarily of tissue-residential/embryonic origin, those resulting from more potent stimulation caused by an infection or severe inflammatory response may originate largely from recruited monocytes (Figure 1). For instance, exposure to certain mucosal stimuli, such as herpes simplex or influenza virus, largely replaces resident AMs with recruited MDMs, while a higher proportion of bona fide resident AMs persist following mucosal exposure to Ad-vectored vaccine or a TLR agonist such as LPS (Kang et al., 2024a; Yao et al., 2018). Indeed, it has been found that airway-resident AMs can temporarily downregulate and re-gain Siglec-F expression accompanied by acquisition and downregulation of CD11b following a single low-dose respiratory LPS exposure (Kang et al., 2024a). It is, however, noteworthy that in some instances of acute lung infection or inflammation, there could be a temporary reduction in the tissue-resident AM population known as the ‘macrophage disappearance reaction’, which reflects the ability of AMs to adapt a different immune phenotype during acute infection and reset their phenotype back to resident AMs in the resolution phase (Aegerter et al., 2022; Janssen et al., 2011; Kang et al., 2024a). Such temporary reduction in tissue-resident AMs as defined by cell surface immunostaining should not be mistaken for bona fide depletion of AMs and its subsequent replenishment by MDMs without other evidence than surface immunostaining.

## Induction of innate immune memory in lung tissue-resident macrophages following systemic immunological exposure

Until recently, prevailing evidence suggested a paradigm of compartmentalization in the development of resting-state innate immune memory, shaped by recent history of immunological imprinting. According to this view, trained hematopoietic progenitors and circulating monocytes are associated with systemic or parenteral exposure to biotic/abiotic factors or vaccines, whereas the innate immune memory in mucosal-resident macrophages at barrier sites is attributed to local biotic/abiotic exposure or vaccination. However, recent findings challenge this notion, revealing that remote exposure to immune stimuli can have far-reaching training effects on innate cells at distant barrier tissue sites, such as the peritoneal cavity and lung (Jeyanathan et al., 2022b). This newly emerged type of immunological action can be referred to as distally imparted local training (Figure 1).

It has been well documented that intravenous BCG (ivBCG) vaccination reprograms hematopoietic stem cells (HSCs) in the bone marrow toward myelopoiesis, fostering innate immune memory in peripheral myeloid cells (Kaufmann et al., 2018). Until recently, it was unclear whether parenteral or subcutaneous BCG (scBCG) vaccination, a non-mucosal route of microbial exposure, could impact lung tissue-resident macrophages (Jeyanathan et al., 2022b). In mice immunized with a low dose of scBCG, AMs display key features of innate immune memory, including increased MHCII expression, enhanced phagocytic capacity, increased glycolysis, and oxidative phosphorylation (Jeyanathan et al., 2022b). Induction of trained AMs occurs in the absence of BCG dissemination to the lung. These trained AMs provide enhanced early protection against *Mycobacterium tuberculosis* (*Mtb*) – the intended target pathogen of the BCG vaccine – preceding adaptive T cell immunity in the lungs, and they also offer protection against heterologous *S. pneumoniae* infection, independent of centrally trained circulating monocytes (Jeyanathan et al., 2022b; Kang et al., 2023). These experimental findings are supported by epidemiological evidence in children and adults showing nonspecific protection by cutaneous BCG vaccination against respiratory infections and mortality (Giamarellos-Bourboulis et al., 2020; Stensballe et al., 2005). Transcriptional reprogramming in these AMs results in increased expression of a defense-ready gene signature, including interferon-associated responses, as well as increased expression of cell cycle markers indicative of proliferation (Jeyanathan et al., 2022b; Mai et al., 2024). Interestingly, a contained intradermal infection of *Mycobacterium tuberculosis* (CMTB) in mice also enhances protection against heterologous *Listeria monocytogenes* infection and lung metastasis of B16 melanoma cells, which is associated with reprogramming of tissue-resident lung macrophages (Nemeth et al., 2020). Like after scBCG immunization, AM remodeling in these mice occurs without *Mtb* dissemination to the lungs. However, ongoing interactions between the immune system and live *Mtb* are essential for maintaining TII, as antibiotic treatment following *Mtb* exposure diminishes protective immunity against secondary infections. Supporting this, inactivated BCG fails to induce innate immune memory in AMs (Jeyanathan et al., 2022b). Remarkably, AM reprogramming persists long after parenteral exposure to either BCG or *Mtb*. This innate immune memory in AMs develops locally within the self-renewing AM population, with a minimal contribution from circulating MDMs, highlighting the role of local signals rather than the involvement of myelopoiesis in the bone marrow. Of note, gross cellularity in the airways of either scBCG or CMTB mice remains unaffected after exposure (Jeyanathan et al., 2022a; Mai et al., 2024).

In contrast to findings from experimental models, AMs isolated from the sputum of BCG-vaccinated individuals display reduced levels of activation markers, such as CD11b and HLA-DR post-vaccination (Koeken et al., 2020). Moreover, these sputum-derived AMs exhibit a lack of response to microbial products, possibly due to a heightened activated state upon isolation. This observation contradicts the known responsiveness of AMs obtained via bronchoalveolar lavage, which typically respond to ex vivo stimulation (Mwandumba et al., 2007). This raises the question of whether this hyperresponsiveness is an inherent feature of sputum-derived AMs, which largely reside in the upper respiratory tract, or if they differ fundamentally from lower respiratory tract AMs in BCG-vaccinated individuals. It is important to note that the immune profile at baseline affects how well innate immune memory develops after BCG vaccination. The vaccine enhances innate immune memory in those with a dormant baseline immune state, rather than providing a broad immune boost across all individuals (Moorlag et al., 2024). Additionally, in humans, the degree of training in peripheral myeloid cells is influenced by the BCG strain (Xu et al., 2024) and even the time of day when BCG is administered (de Bree et al., 2020). Future studies should investigate how these factors affect the induction of innate immune memory in lung-resident macrophages following BCG vaccination. Further understanding of the relationship of cutaneous BCG vaccination to lung-resident macrophage training and its heterogeneity will shed light on the innate immune mechanisms underlying TB resistance observed in a proportion of *Mtb*-exposed humans (Koeken et al., 2019).

Unlike the IFN-γ-dependent induction of innate immune memory in HSCs in the bone marrow following ivBCG vaccination (Kaufmann et al., 2018), the induction of innate immune memory in AMs by scBCG occurs independently of IFN-γ in murine models (Jeyanathan et al., 2022b). Instead, innate immune memory in AMs after scBCG is linked to gut microbiota dysbiosis and elevated levels of short-chain fatty acids (SCFA), carnitine, and butyrate in the intestine, circulation, and lung tissue (Jeyanathan et al., 2022b). Microbiota transplantation or SCFA supplementation via drinking water in naïve mice replicates these effects, indicating a causal link between BCG-induced gut microbiota changes and the development of memory AMs. This points to a gut-lung axis in the induction of innate immune memory in AMs (Roquilly and Villadangos, 2022). In this regard, intestinal colonization by segmented filamentous bacteria (SFB) leads to reprogramming of AMs, enhancing their ability to control respiratory viruses and reduce disease severity (Ngo et al., 2024). However, the specific signal from SFB colonization that drives AM reprogramming remains unclear. The aforementioned findings highlight the role of fatty acids and their metabolic products in reprogramming lung-resident macrophages. Lipid mediators – bioactive signaling molecules generated by the oxidation of polyunsaturated fatty acids like arachidonic acid (AA) – serve as key signaling agents in establishing innate immune memory in monocytes following scBCG vaccination in humans (Ferreira et al., 2023). Monocytes trained in vitro with BCG exhibit elevated levels of metabolic products from two major enzymatic pathways: cyclooxygenase and lipoxygenase, which metabolize AA into eicosanoids. To this end, AMs are a significant source of bioactive lipids that act as key sensors in regulating pulmonary immunity during viral infection (Pernet et al., 2024). However, it remains to be determined whether systemic exposure to immune stimuli induces production of lipid mediators by AMs and plays a role in memory formation and maintenance. Understanding the interaction between lipid mediators and pulmonary macrophages could have important implications for developing therapeutic strategies against respiratory infections.

Although numerous studies have established that parenteral exposure to defined microbial products trains bone marrow and circulating myeloid cells, its influence on tissue-resident macrophages at distal barrier sites remains relatively underexplored. Recent evidence demonstrates that parenteral β-glucan exposure in mice significantly impacts lung-resident macrophages by enhancing their efferocytic capacity and providing critical survival signals to epithelial cells, thereby increasing resilience to bleomycin-induced tissue injury (Kang et al., 2024b). While the precise role of monocyte myelopoiesis in contributing to the lung tissue-resident memory macrophage pool remains unclear, β-glucan treatment promotes the recruitment of apoptotic neutrophils to the lung, correlating with histone modifications in AMs. These trained AMs exhibit upregulated expression of *Il4ra* and *Il13ra* gene transcripts and produce elevated levels of the lipid mediator resolvin D1 (RvD1). Notably, alveolar epithelial cells cultured in conditioned media from these trained AMs display enhanced expression of SIRT1, which is associated with reduced epithelial apoptosis (Kang et al., 2024b). These findings suggest that parenteral exposure to microbial products can also exert significant effects on tissue-resident macrophages, highlighting a novel mechanism for enhancing epithelial cell protection in barrier tissues.

Studies have further shown that acute tissue injury can induce innate immunity in distal barrier tissue sites of mice. AMs exhibit increased proliferation, heightened expression of epidermal growth factor receptor (EGFR), and increased epidermal growth factors in the lung following myocardial infarction (MI) (Hoyer et al., 2019). The AM population remains stable post-MI, with initial priming occurring independent of circulating monocytes, but rather through IFN-γ produced by lung-recruited NK cells. Importantly, AMs show enhanced phagocytic activity and provide enhanced pathogen clearance during subsequent respiratory bacterial infection, such as those caused by *S. pneumoniae*, while they contribute to inflammatory lung injury due to increased EGFR signaling (Hoyer et al., 2019). Interestingly, the pathogen clearance benefits seen after MI are not observed following stroke or sepsis, despite similar alterations in AMs. Although whether such distal injury-induced changes in AMs remains unclear, this study raises the possibility that lung-resident macrophages may undergo changes in response to a wide array of immunological events occurring at other tissue sites.

Current evidence suggests that monocytes play a relatively minor role in the development and maintenance of innate immune memory in lung tissue-resident macrophages, particularly AMs, following remote exposure to immune stimuli. This contrasts with observations following local exposure, where the degree of inflammation can lead to various outcomes, from no replacement to partial or complete replacement of lung tissue-resident macrophages by circulating monocytes (Figure 1). In murine studies, AMs are seeded during embryonic development and can self-replenish independent of circulating monocytes throughout adulthood (Guilliams and Svedberg, 2021). However, less is known regarding the ontogeny of airway macrophages in the human lung. A recent study demonstrated that, in lung transplant patients, the majority of airway macrophages were replaced by circulating monocytes, which differentiated into mature AMs (Byrne et al., 2020). This suggests that the ontogeny of human AM population may be more dynamic and less stable than murine counterparts and emphasizes the importance of delineating the origin of AMs in human and murine lungs (Byrne et al., 2020). Furthermore, this raises the question as to how applicable murine models are to humans, and the data should be interpreted with caution. Since the majority of murine models used are kept in SPF conditions and are inbred, they do not accurately recapitulate the respiratory mucosal environment in humans. Thus, a better model to aid in the translation of murine models to humans would be to adopt a mouse model that closely resembles humans, including the use of pet store or deer mice (Hamilton et al., 2020; Kitching and Ooi, 2016). There also remains a gap in our understanding of how both local and remote exposure to immune stimuli affects other lung tissue-resident macrophage populations besides AMs, including IMs. However, the distinct microenvironment where AMs reside, which are isolated from circulation during homeostasis (Hashimoto et al., 2013), unlike IMs, may explain why the memory AM population remains intact without significant monocyte contribution following either a local or remote immunological event.

The field of remote immune modulation of lung-resident macrophages represents a promising frontier for both basic and clinical research. Understanding how innate immune memory forms in barrier tissues, in the context of interorgan immune communication via host- and natural microbiota-derived signals, has the potential to revolutionize vaccine strategies and therapies for lung diseases and enhance vaccine strategies against respiratory infections. Moving forward, research must delve into the specific molecular mechanisms involved and carefully evaluate the risks of respiratory mucosal and systemic immune approaches, especially in the context of autoimmune diseases.

## TII and immunological outcomes in the lung

In the past decade, systemic – and more recently, local – immunological exposure to various microbial components, vaccines, and infections has been shown to alter the innate immune cell compartment, specifically within the tissue-resident macrophage population at mucosal sites (Netea et al., 2016; Xing et al., 2020). It is known that such immune or inflammatory memory within the innate immune cell compartment can result in a strengthened immune response leading to enhanced innate immune protection against either a homologous or heterologous pathogen/entity (Table 1). As described in the Introduction, the altered innate immune protective outcome resulting from innate immune memory is referred to as TII. Since locally or centrally induced TII is capable of heightened innate immune responses and protection against a broad range of pathogens and entities in the lung, its protective mechanisms may vary widely and are thus worthy of separate considerations.

**Table 1.** The outcome of systemic and/or local immunological exposure-induced trained innate immunity (TII) in different pathological conditions.

Barrier tissue site	Pathology	Outcome	References
Lung	Pneumonia	Protection	Kang et al., 2024a; Kang et al., 2023; Yao et al., 2018; Zahalka et al., 2022
		Promotes disease	Roquilly et al., 2020
	TB	Protection	Bickett et al., 2020; D’Agostino et al., 2020; Jeyanathan et al., 2022b; Moorlag et al., 2020
	Influenza	Protection	Kaufmann et al., 2022; Khan et al., 2025; Lercher et al., 2024
		Promotes disease	Li et al., 2022a
	COVID-19	Protection	Afkhami et al., 2022; Hilligan et al., 2022; Oyesola et al., 2023; Zhang et al., 2022
	Helminth	Protection	Chen et al., 2014
	Lung cancer	Protection	Wang et al., 2023
Genital tract	Bladder cancer	Protection	Daman et al., 2025; Jurado et al., 2025
Skin	Staph infection	Protection	Chan et al., 2018; Chan et al., 2017; Feuerstein et al., 2020
GI tract	Salmonella	Protection	Ahrends et al., 2021
Peritoneal cavity	Staph infection	Protection	Yoshida et al., 2015
	Peritonitis	Protection	Ciarlo et al., 2020
	Endometriosis	Protection	Jeljeli et al., 2020
Oral cavity	Periodontitis	Promotes disease	Li et al., 2022b

### TII against respiratory bacterial infections

Both systemic and local exposure to vaccines and microbial stimuli can induce TII in lung-resident macrophages against heterologous pneumococcal pneumonia, leading to enhanced protection (Kang et al., 2024a; Kang et al., 2023; Yao et al., 2018; Zahalka et al., 2022). Such enhanced antibacterial TII against acute pulmonary *S. pneumoniae* infection observed in murine models was driven by heightened bactericidal activity of trained AMs, increased chemokine responses, and accelerated pulmonary neutrophilia during the early phases of bacterial infection. This led to improved clinical disease outcomes and accelerated bacterial clearance, improving overall survival. This TII-mediated enhanced protection against pneumococcal pneumonia was independent of circulating monocytes. Pneumococcal pneumonia is a major infectious respiratory disease that mainly affects young children and the elderly (Brooks and Mias, 2018). Current human serotype-specific polysaccharide vaccines are designed for intramuscular administration and aim to elicit adaptive antibody responses and may select for the dominance of new strains/serotypes capable of immune evasion. New vaccination strategies must be designed to overcome this limitation and provide serotype-independent protection; one such approach involves complementing such adaptive immune responses with the induction of innate immune memory and TII.

Local lung β-glucan administration in mice induces low-grade pulmonary inflammation and trained ApoE^+^CD11b^+^ AMs, which provide protection against *Legionella pneumophila* infection and lung fibrosis (Theobald et al., 2024). Such enhanced heterologous protection was attributed to the regulatory role of ApoE^+^CD11b^+^ AMs that produced significantly greater levels of pro-resolving lipid mediators allowing for reduced severity of bacterial infection and inflammation. However, it is important to note that respiratory bacterial infection drives recruitment of various innate immune cells to the lung, and these ancillary innate cell populations may be imprinted in parallel, altering their responsiveness to pneumonia and should not be overlooked.

Respiratory mucosal vaccination with a recombinant Ad-vectored TB vaccine in mice induced trained AMs, which protect against the intended target *Mtb* infection by controlling bacterial growth during the early stages of infection and improving disease outcomes (D’Agostino et al., 2020). Akin to this, systemic BCG-induced trained AMs also protected against acute *Mtb* infection (Bickett et al., 2020; Jeyanathan et al., 2022b). In both cases, such TII linked to enhanced homologous protection against *Mtb* was independent of circulating monocytes and dependent on trained AMs. In addition, systemic β-glucan-induced TII has been linked to protection against pulmonary *Mtb* infection via IL-1 signaling, but the contribution of trained tissue-resident macrophages in such TII against TB was not examined in this experimental model (Moorlag et al., 2020). The IL-1 signaling axis has been widely described as a critical mediator of TII, and the availability of pharmacological antagonists, such as anakinra, warrants its inclusion as a mechanistic target for studies investigating innate immune memory.

### TII against respiratory viral infections

Respiratory viral infections, including IAV and SARS-CoV-2, are estimated to infect up to a billion people annually, causing millions of deaths. Over the course of various pandemics caused by these viral pathogens, current vaccination strategies that elicit strain-specific immunity failed to protect against rapidly emerging variants, as they evade antigen-dependent adaptive immunity. In this regard, emerging evidence suggests that locally or centrally induced TII can be exploited to offer enhanced innate immune protection against acute respiratory viral infection in the lung via enhancing disease tolerance (King and Divangahi, 2019; McCarville and Ayres, 2018). Enhanced disease tolerance is a host defense mechanism that operates by limiting tissue immunopathology for improved disease outcomes and host survival, without significant changes in microbial clearance or the magnitude of infection. Enhanced disease tolerance has been observed in a number of infectious disease models, including those of acute respiratory viral infection (Afkhami et al., 2022; Khan et al., 2025; Nahrendorf et al., 2021; Pernet et al., 2019). In particular, respiratory or parenteral induction of TII has been observed to protect against acute SARS-CoV-2 or influenza infection in the lung via enhanced viral disease tolerance (Afkhami et al., 2022; Khan et al., 2025). Of importance, such enhanced disease tolerance can be accomplished entirely independent of adaptive immune responses (Afkhami et al., 2022). In keeping with these preclinical observations, human vaccination with BNT162b2 mRNA vaccine induced long-term transcriptional changes in circulating PBMCs, which led to a dampened response upon ex vivo re-stimulation with a wide range of viral ligands (Föhse et al., 2023), implying a likely phenotype of enhanced viral disease tolerance.

On the other hand, a number of studies have demonstrated that locally or centrally induced TII is able to offer enhanced protection against acute respiratory viral infection via accelerated viral clearance and reduced tissue immunopathology (Brandi et al., 2022; Hilligan et al., 2022; Kaufmann et al., 2022; Lee et al., 2024; Lercher et al., 2024; Tran et al., 2024; Zhang et al., 2022). In such studies, it is most likely that mitigated tissue immunopathology is attributable to reduced viral burden. However, one such study suggests that the disease-tolerizing mechanism may also have played a role (Hilligan et al., 2022). While the precise mechanisms driving enhanced innate protection by TII seen in these studies remain to be fully elucidated, recent work suggests that SARS-CoV-2 infection epigenetically reprograms airway-resident macrophages, enhancing the expression of type I interferon transcription factors and other antiviral genes (Lercher et al., 2024). In contrast, vaccine-induced TII-enhanced protection against SARS-CoV-2 infection was mediated via crosstalk between adaptive and innate immune memory responses in experimental models (Lee et al., 2024; Tran et al., 2024). Such protection was attributed to IFN-γ and T cell responses that bolstered antiviral immunity of AMs. Moreover, this created a feedback loop between imprinted tissue myeloid and epithelial cells in the lung, yielding enhanced innate antiviral immunity (Lee et al., 2024).

Due to the limited understanding of training in airway-resident macrophages following local or systemic immunological exposure and TII against respiratory viral infections, their contribution to protection relative to centrally trained monocytes remains unclear and warrants further investigation. Likewise, a better understanding of the longevity of heterologous innate protection mediated by various stimuli-induced TII in the lung is required. Thus, the innate immune functional outcomes and mechanisms of respiratory mucosal Ad vaccine-induced TII may vary, depending on the nature of the heterologous challenge. We have found that local Ad-vectored vaccine-induced TII provides enhanced protection against SARS-CoV-2 infection, independent of lung viral burden via enhanced disease tolerance and regulation of inflammatory responses in the lung of mice (unpublished). Interestingly, although such respiratory mucosal Ad-induced TII provides enhanced protection against both SARS-CoV-2 and pneumococcal infection, a recent study shows that local LPS exposure-induced TII provides robust protection against pneumonia but extends minimal protection against acute respiratory SARS-CoV-2 infection (Kang et al., 2024a). While the exact mechanisms that explain such differential protection remain to be investigated, these findings suggest that locally induced TII is not created equal and that the operating mechanisms may differ according to microbial target cell types (extracellular bacteria target phagocytic macrophages, whereas viruses primarily infect epithelial cells).

### TII and lung tissue repair

Less is known regarding TII against lung injury, specifically how TII regulates and resolves inflammation and tissue injury resulting from either infectious or noninfectious events. Following exposure to different microbial stimuli, trained tissue-resident macrophages exhibit increased efferocytosis, leading to better lung injury resolution (Chakraborty et al., 2023; Kang et al., 2024b). Trained AMs produce soluble mediators along with enhanced efferocytosis and increased levels of SIRT1 in lung epithelial cells of mice, leading to decreased apoptosis and the restoration of tissue homeostasis. However, the role of lipid mediators in TII against tissue injury remains to be investigated. Understanding the interaction between pro-resolving mediators produced by imprinted tissue-resident macrophages and TII in regulating tissue injury and repair could have important implications for developing therapeutic strategies against lung injury and fibrosis. On the other hand, recent findings demonstrate that systemic administration of β-glucan reprograms AMs via transcriptional and metabolic modifications and is independent of dectin-1 in murine models (Prevel et al., 2025). However, such trained AMs exacerbate lung injury following secondary exposure to LPS or poly(I:C), leading to severe acute lung injury. Thus, shedding light on the potential deleterious effects of TII on tissue injury requires further investigation.

### T2 immunity and TII in helminth infection

 Recent studies have shown that innate immune memory and TII in circulating monocytes, epithelial cells, and, to a lesser extent, tissue-resident macrophages, can also be linked to T2 inflammation in cases of allergic asthma (Hartung and Esser-von Bieren, 2022; Lechner et al., 2022). While the mechanisms of TII against respiratory viral and bacterial pathogens are relatively well understood, the mechanistic underpinnings and consequences of TII for other diseases, including cancer, cardiovascular disease, and chronic inflammatory conditions, are only beginning to emerge. This is particularly true for T2 immunity, which plays an important role in protection against parasitic infections and in the pathogenesis of allergy and asthma. Helminth infection with *Nippostrongylus brasiliensis* (*Nb*) imprinted macrophages and induced TII linked to enhanced protection against secondary *Nb* infection (Chen et al., 2014). Such protection was dependent on crosstalk between neutrophils and imprinted macrophages of mice, which led to efficient killing of *Nb* larvae. A greater proportion of AMs and IMs isolated from *Nb* infection-imprinted mice displayed an Arginase 1^+^, PLDL2^+^, and CD301^+^ alternatively activated phenotype, leading to expedient nematode clearance upon rechallenge (Chen et al., 2014). More recently, prior *Nb* infection induced lung remodeling and TII, which protected against heterologous acute respiratory SARS-CoV-2 infection, but this was notably not recapitulated following primary infection with *Heligmosomoides polygyrus*, indicating that local pulmonary inflammatory cues are required (Oyesola et al., 2023). *Nb*-imprinted tissue-resident macrophages displayed a T2 profile and increased production of CD8^+^ T cell-recruiting chemokines. This resulted in heightened activation and recruitment of CD8^+^ T cells to the lung following infection (Oyesola et al., 2023). This illustrates how T2 inflammation-induced TII in the lung can enhance viral clearance and limit disease severity during heterologous pulmonary viral infection, which is mediated through indirect interactions between tissue-resident macrophages and T cells. Not much is known regarding resolution and repair in T2 immunity and associated TII. However, tissue remodeling factors were upregulated in helminth-primed macrophages and are generally associated with resolution of inflammation (Oyesola et al., 2023). Thus, activation of these macrophages may promote resolution and repair of the lung tissue following heterologous SARS-CoV-2 infection.

### TII against respiratory mucosal cancer

Recent work has shown that trained AMs can provide long-term antitumor immunity within the lungs. Infection with wild-type IAV led to trained AMs characterized by persistent epigenetic, metabolic, and phenotypic changes, such as enhanced production of proinflammatory cytokines IL-1β, IL-6, and TNF (Wang et al., 2023). In a murine model of B16 melanoma metastatic lung cancer, IAV infection-trained AMs conferred antitumor TII that persisted months following acute infection, characterized by enhanced tumor cell phagocytosis and cytotoxicity. Importantly, these trained AMs were epigenetically resistant to suppressive signals from the tumor microenvironment that promoted immune-suppressive signatures in uninfected control animals. Mechanistically, training was independent of T cell immunity, but reliant on NK cell-derived IFN-γ (Wang et al., 2023). Although such observations highlight a novel facet of TII at the respiratory barrier in enhancing protection against pulmonary metastatic disease, it is important to highlight the minimal improvement in survival – potentially attributed to the aggressiveness of the B16 cancer model. Perhaps, it is not entirely surprising to see the documented observations on TII-mediated protection against respiratory mucosal cancer, given that BCG has long been used in humans as a treatment for noninvasive cancer in a different barrier tissue site – the bladder, for many years. More recently, BCG-induced anti-bladder tumor TII was observed through its reprogramming of HSCs in the bone marrow of mice (Daman et al., 2025) and administration of both BCG and β-glucan-induced robust antitumor TII via enhanced granulopoiesis and trained neutrophils (Jurado et al., 2025). Nonetheless, it would be of interest to consider whether the induction of analogous training – such as that endowed by inhaled Ad vaccines – may strengthen on-target antitumor immunity from the adaptive branch, and whether these can be utilized to strengthen current standard-of-care approaches for malignancies.

### Maladaptive TII and inflammatory conditions

Under physiological conditions, TII can be beneficial against heterologous pathogenic infections, lung injury, tumors, and Th2 immunity, which is followed by the resolution of inflammation and restoration of homeostasis (Merlo Pich et al., 2024). However, in some cases, dysregulation or chronic activation of the innate immune system can contribute to maladaptive TII and perpetuating inflammatory diseases (Li et al., 2022b; Table 1). Epigenetic modifications and metabolic rewiring are the underlying basis in the development and maintenance of innate immune memory, and some epigenetic features can persist from days to months following clearance of the initial insult and can be quickly activated following secondary insult (Niec et al., 2021).

Long after primary pneumonia infection and resolution of inflammation, AMs are impaired and exhibit poor phagocytic capacity following subsequent bacterial infection (Roquilly et al., 2020). Immunosuppressive signals produced locally in the lung microenvironment tolerize resident AMs, rendering them ‘paralyzed AMs’. These tolerized AMs persisted long after resolution of inflammation and were not monocyte-derived but embryonic-derived self-renewing resident AMs. SIRPα production in the lung microenvironment played a key role in the establishment of the immunosuppressive state and mediated induction of tolerance in AMs, not the direct interaction between AMs and the pathogen (Roquilly et al., 2020). In this case, paralysis was restricted to tissue-resident macrophages in the lung; thus, it is important to consider inflammatory responses that potentially extend more widely than just the lung and cause multiorgan damage and inflammatory adaptation at other barrier tissue sites. While it is known that many chronic inflammatory diseases are driven by inflammatory myeloid cells, the impact of tissue-resident macrophages is less studied. Maladaptive TII in bone marrow progenitors has been linked to inflammatory comorbidities and plays a role in periodontitis and arthritis (Li et al., 2022b). Periodontitis-induced systemic inflammation led to epigenetic rewiring of HSPCs and sustained enhancement of production of myeloid cells with increased inflammatory responses, thus increasing susceptibility to subsequent inflammatory conditions. The induction of maladaptive TII was dependent on IL-1 signaling in HSPCs (Li et al., 2022b). Furthermore, following primary IAV infection, there was a substantial loss of embryonic-derived AMs, which were replenished via self-renewal of survivors and generation of monocyte-derived AMs (Li et al., 2022a). Several months post-primary viral infection and viral clearance, monocyte-derived AMs outcompeted embryonic-derived AMs due to their increased glycolytic and proliferative capacity. However, the presence of monocyte-derived AMs resulted in severe pathology following recurrent IAV infection due to their more pronounced proinflammatory profile. With age, embryonic-derived AMs were gradually replaced by monocyte-derived AMs and contributed to increased disease severity to IAV infection (Li et al., 2022a). This suggests that the ontogeny, rather than the training agent/stimuli, of AMs may determine their long-term phenotype and reaction to respiratory viral infection.

## Induction of innate immune memory and TII in tissue-resident macrophages at other barrier tissue sites

Beyond the lung, tissue-resident memory macrophages and TII are also induced at other barrier tissues, such as the skin, gut, and peritoneal cavity. This induction enhances innate immune responses, allowing these tissues to mount more rapid and effective TII against subsequent unrelated pathogens.

### TII at the skin

As the body’s largest barrier organ, the skin is constantly exposed to an array of microbes, including commensals and pathogens. Recent evidence indicates that this interface can harbor tissue-resident innate immune cells capable of developing innate immune memory and associated TII (Chan et al., 2017; Feuerstein et al., 2020). Skin-resident trained macrophages can be programmed to respond more swiftly and effectively to subsequent microbial challenges, yet the extent of cross-protection and compartmentalization remains an active area of investigation. This section examines the interplay between TII and *Staphylococcus aureus* (*S. aureus*), the most common cause of skin and soft tissue infections, and discusses how tissue-resident memory macrophages, metabolic reprogramming, and microbial factors converge to shape immune outcomes at this critical barrier site.

In a murine model of sequential *S. aureus* infection, primary infection induces local TII and reduces bacterial burden and lesion size following subsequent homologous challenge (Chan et al., 2018; Chan et al., 2017; Feuerstein et al., 2020). This protection was highly compartmentalized as protection was not seen on the contralateral side. Furthermore, the removal of macrophages via clodronate liposome treatment ablated the phenotype (Feuerstein et al., 2020). Importantly, the contribution from adaptive immune cells and circulating monocytes was ruled out, emphasizing the induction of memory in tissue-resident innate immune cells.

While sequential *S. aureus* infection-associated TII can lead to more rapid resolution of secondary abscesses, low-pathogenicity *S. aureus* small colony variants (SCV) commonly cause chronic infections and can switch to an antimicrobial-resistant persister phenotype (Lin et al., 2024). Intriguingly, attenuated infection from a Δ*hemB* SCV failed to elicit an equivalent trained immune response compared to wild-type *S. aureus* infection, despite more potent induction of glycolysis in human PBMCs, THP-1 cells, and keratinocytes (Wong Fok Lung et al., 2020). Heightened glycolysis was associated with increased expression of succinate dehydrogenase, leading to increased intracellular fumarate concentrations, a metabolite critical for the development of TII in monocytes. Unfortunately, while this study failed to investigate the metabolic fate of bona fide skin tissue-resident macrophages during SCV infection, it demonstrates a role for bacterial modulation of host metabolism as an inhibitor of TII. Together, these findings underscore the central role of TII in protecting the skin against infection, while also highlighting significant gaps in our understanding. Although targeting host and microbial metabolic pathways holds promise for enhancing antibacterial efficacy, it remains unclear how effectively such approaches will protect against antimicrobial-resistant strains or emerging viral threats. Future studies must disentangle the complex interplay between microbial virulence factors, metabolic reprogramming in tissue-resident macrophages, and the broader immune network within the skin.

### TII at the gastrointestinal tract

Barrier tissues, such as the intestine, balance nutrient absorption with epithelial integrity, inflammation, blood flow, and innervation to control smooth muscle contractility. Consequentially, gut tissue-resident macrophages fill diverse subtissular niches in the epithelium, lamina propria, submucosa, and muscularis externa, all with unique contributions from circulating monocytes (Delfini et al., 2022). Distal from the lumen, relatively non-motile muscularis externa macrophages defend nerve bundles and blood vessels from infection (De Schepper et al., 2018).

Infecting mice with *Yersinia pseudotuberculosis* abrogated neuronal loss during secondary heterologous infection with *Salmonella typhimurium* (SpiB) in the absence of accelerated bacterial clearance (Ahrends et al., 2021). In contrast to enhanced pathogen clearance as a hallmark of adaptive immune memory, innate immune memory against stressors may instead manifest as disease tolerance to diminish inflammatory damage and promote tissue maintenance/recovery (Ayres, 2020), as aforementioned in the context of viral disease tolerance in the lung. Notably, neuroprotection against SpiB challenge was observed in pet-store mice in the absence of a primary infection, indicating that immune history, microbiota, and genetics contribute to tissue tolerance. This highlights yet another gap in our knowledge as diet and microbiota perturbations due to infection/vaccination status, or therapeutics (e.g. antibiotics/metabolic modulators), can affect the production of soluble innate immune training agents that may act locally or even distally through the skin-gut and gut-lung axes (Christ et al., 2018; Jeyanathan et al., 2022b; Ngo et al., 2024; Sencio et al., 2020).

Similarly, muscularis macrophage-mediated neuronal protection against secondary SpiB infection was observed following primary *Strongyloides venezulenesis* helminth infection. This protective mechanism is through a Th2 and eosinophil-dependent manner, underscoring the complexity of immune cell interactions (Ahrends et al., 2021). Therefore, a systematic approach is expected to address the role of TII. Not only must the roles of central training and adaptive immune cells be parsed apart from resident innate cells in peripheral sites, but recruited circulating monocytes may indirectly provide local TII by educating resident immune and/or stromal cells analogous to Ad-vectored vaccine-derived CD8^+^ T cells in inducing memory AMs (Yao et al., 2018).

In addition to acute gastrointestinal infections conferring heterologous protection against secondary challenge, helminth infection can exert both positive and negative effects during viral co-infection (Desai et al., 2021; Furze et al., 2006; McFarlane et al., 2017; Osborne et al., 2014; Rolot et al., 2018). However, further investigation is required to determine if immune perturbations from these infections impart long-lasting alterations or bona fide memory to innate immune cells or are merely a byproduct of coincident infections. Furthermore, it is unknown if training of gut tissue-resident macrophages or hematopoietic progenitors occurs following gastric virus infection. This is critical due to the real-world epidemiological effects observed following oral polio vaccination. The oral polio vaccine (OPV), a live-attenuated poliovirus, nonspecifically reduces gastrointestinal infections, respiratory infections, and all-cause mortality, particularly in males (Andersen et al., 2018; Contreras, 1989; Contreras, 1974; Nielsen et al., 2021; Sørup et al., 2016; Welaga et al., 2017). Due to reduced morbidity locally within the gastrointestinal tract and distally at the respiratory mucosa, OPV may induce a combination of local intestinal training, production of soluble mediators with distal mechanisms of action, and/or central training. However, the exact cells that involve OPV-induced TII – whether they are tissue-resident macrophages, inflammatory MDMs, or intestinal stromal cells – remain unknown.

Eliciting a tolerogenic state in gut-resident macrophages could offer an appealing avenue to treat inflammatory bowel disease, colitis, and Crohn’s disease. Each disease is characterized by chronic inflammation, and all have well-documented contributions from inflammatory monocytes (Delfini et al., 2022). An expanding body of work suggests that recruited monocytes that develop tissue residency following an insult retain a heightened proinflammatory phenotype relative to bona fide tissue-resident counterparts (Guilliams and Svedberg, 2021). Some gut-resident macrophage populations, such as those in the lamina propria, are constantly replenished from monocytes, suggesting that the replenishing monocytes may potentially be involved in these conditions. Discretion is required to select methods sensitive enough to delineate contributions from macrophages that occupy distinct subtissular niches within tissues such as the gut, as they play diverse roles in tissue homeostasis. Detailed longitudinal fate-mapping studies are required to observe how resident macrophage populations change in response to inflammation and the lasting ‘immunological scars’ after inflammation constitute a complex phenomenon described as the ‘macrophage disturbance of homeostasis reaction’ (Salm et al., 2023; Zhao et al., 2024).

### TII within the peritoneal cavity

At homeostasis, most peritoneal macrophages (pMacs) consist of self-sustaining Gata6^+^, fetal liver-derived large peritoneal macrophages (LPMs) maintained by omentum-derived AA and monocyte-derived small peritoneal macrophages (SPMs) (Salm et al., 2023). LPMs are among the first cells to respond to peritonitis that may be caused by intestinal perforation and subsequent infection with gut bacteria. Upon systemic exposure to LPS, pMacs phosphorylate the anti-inflammatory transcription factor Atf7, consequently reducing repressive histone marks and leading to enhanced protection against secondary heterologous *S. aureus* infection in murine models (Yoshida et al., 2015). Of note, this pathway was enriched following systemic training from β-glucan, but not peptidoglycan or imiquimod, suggesting that TLR ligands from bacteria, yeast, or viruses induce distinct epigenetic changes within innate immune cells (Yoshida et al., 2015). Furthermore, intraperitoneal β-glucan-induced TII altered the pMac composition with a greater proportion of bactericidal SPM persisting long after the initial insult and protecting against *Escherichia coli* peritonitis (Ciarlo et al., 2020). Since TII results from the long-term imprinting of innate immune cells mediated by epigenetic and metabolic alterations, the macrophage disappearance reaction and replacement of embryonic-derived macrophages with MDMs represent a distinct physiological process that requires further investigation (Salm et al., 2023).

In a murine model of endometriosis, priming via systemic BCG vaccination increased inflammatory infiltrates and lesion size, while tolerization via repeated doses of LPS decreased fibrosis and lesion weight (Jeljeli et al., 2020). The phenotype was dependent on recruited circulating monocytes as BCG-primed pMacs responded to endometriotic cells through the production of monocytic chemokines. While reduced severity of endometriosis was corroborated in a human cohort with a history of severe gynecological Gram-negative bacterial infections, the mouse model failed to accurately recapitulate TII, as the final priming dose of BCG or LPS was given only 2 days prior to endometriosis surgery (Jeljeli et al., 2020). Similarly, TII induced by systemic injection of oroxylin A (Yin et al., 2024), β-glucan derivatives (Mao et al., 2025; Pan et al., 2022), and *Lactobacillus plantarum* (Santecchia et al., 2019) protected against diverse nonspecific pathogens. However, each of these murine models better represents priming than bona fide TII due to the inadequate time interval between the initial stimulus and secondary challenge. Nonetheless, the discovery of novel innate immune training agents is vital, and each of these studies requires further investigation regarding the duration of the protective effects.

There is limited knowledge on the long-term changes in the phenotype and functionality of tissue-resident macrophages in the peritoneal cavity, as most studies examined only the acute changes. However, more recently, besides induction of memory AMs in the lung, scBCG vaccination also had a long-term global training effect on pMacs due to a connection between the peritoneal cavity and other organs within the cavity, including the gut (Jeyanathan et al., 2022b). Such BCG pMacs exhibited a trained phenotype with increased expression of MHCII and increased production of proinflammatory cytokines upon ex vivo restimulation. The development of memory pMacs was dependent on the dissemination of live BCG bacilli to the peritoneal cavity, as inactivated BCG failed to induce trained pMacs. Furthermore, the induction of memory pMacs was independent of circulating monocytes and led to TII against heterologous bacterial infection (unpublished).

## Inflammatory memory and TII of nonimmune epithelial cells at barrier tissue sites

Compared to our knowledge in innate immune memory and TII associated with innate immune cells and their progenitors, limited information is available for nonimmune innate tissue structural cells. However, recent evidence indicates the acquisition of long-lasting innate inflammatory memory and TII in nonimmune or structural cells in the lung (Hamada et al., 2018). Furthermore, inflammatory memory has been shown in stem cells within other barrier tissue sites, such as the skin (Cheng et al., 2023). Like trained innate immune cells, such trained nonimmune cells are also capable of TII against subsequent heterologous pathogen exposure.

Indeed, in addition to tissue-resident macrophages in the lung, bronchial epithelial cells (BECs) participate in innate host defense and are among the first cells to encounter inhaled respiratory pathogens (Bigot et al., 2025). Recently, flagellin exposure in mice has been shown to modify the inflammatory phenotype of BECs following secondary exposure to unrelated immune stimuli via sustained epigenetic modifications (Bigot et al., 2025; Bigot et al., 2019). Pre-exposure of BECs with *Pseudomonas aeruginosa* flagellin in vitro induced histone modifications altering gene expression leading to modulation of the inflammatory response to subsequent heterologous exposure to immune stimuli. Such memory BECs produced elevated levels of cytokines IL-8 and IL-6 following secondary exposure to a live fungal pathogen; in contrast, they exhibited a decreased inflammatory response against subsequent LPS stimulation (Bigot et al., 2019).

On the other hand, recent advances in the field showed enhanced wound healing after the skin of mice was exposed to imiquimod, a topical immune response modifier, suggesting skin epithelial stem cells can retain inflammatory memory in response to tissue damage (Cheng et al., 2023; Naik et al., 2017). In a model of skin inflammation, epithelial stem cells demonstrated inflammatory memory associated with long-lasting chromatin modifications acquired following tissue damage (Naik et al., 2017). Such inflammatory-induced memory skin epithelial stem cells display a heightened response to subsequent stressors associated with enhanced wound healing. However, the mechanisms underlying such inflammation-induced rewiring of skin epithelial stem cells require further investigation. Inflammation-experienced memory skin epithelial stem cells may underlie recurrent skin inflammation displayed in autoimmune skin disorders and may have implications for future therapeutic strategies.

Besides lung and skin epithelial cells, the limited but emerging evidence also suggests the epithelial cells at other barrier tissues, such as the intestine, to acquire inflammatory memory and TII (Chen et al., 2024). Specifically, this was demonstrated in intestinal tuft cells primarily mediated by IL-25 in a murine enteroviral infection model. While the data obtained largely from in vitro models also suggest the induction of inflammatory memory in other structural cells, such as smooth muscle cells and fibroblasts, further in vivo investigation is required.

## TII-based vaccine and immunotherapeutic strategies

**Figure 2.**
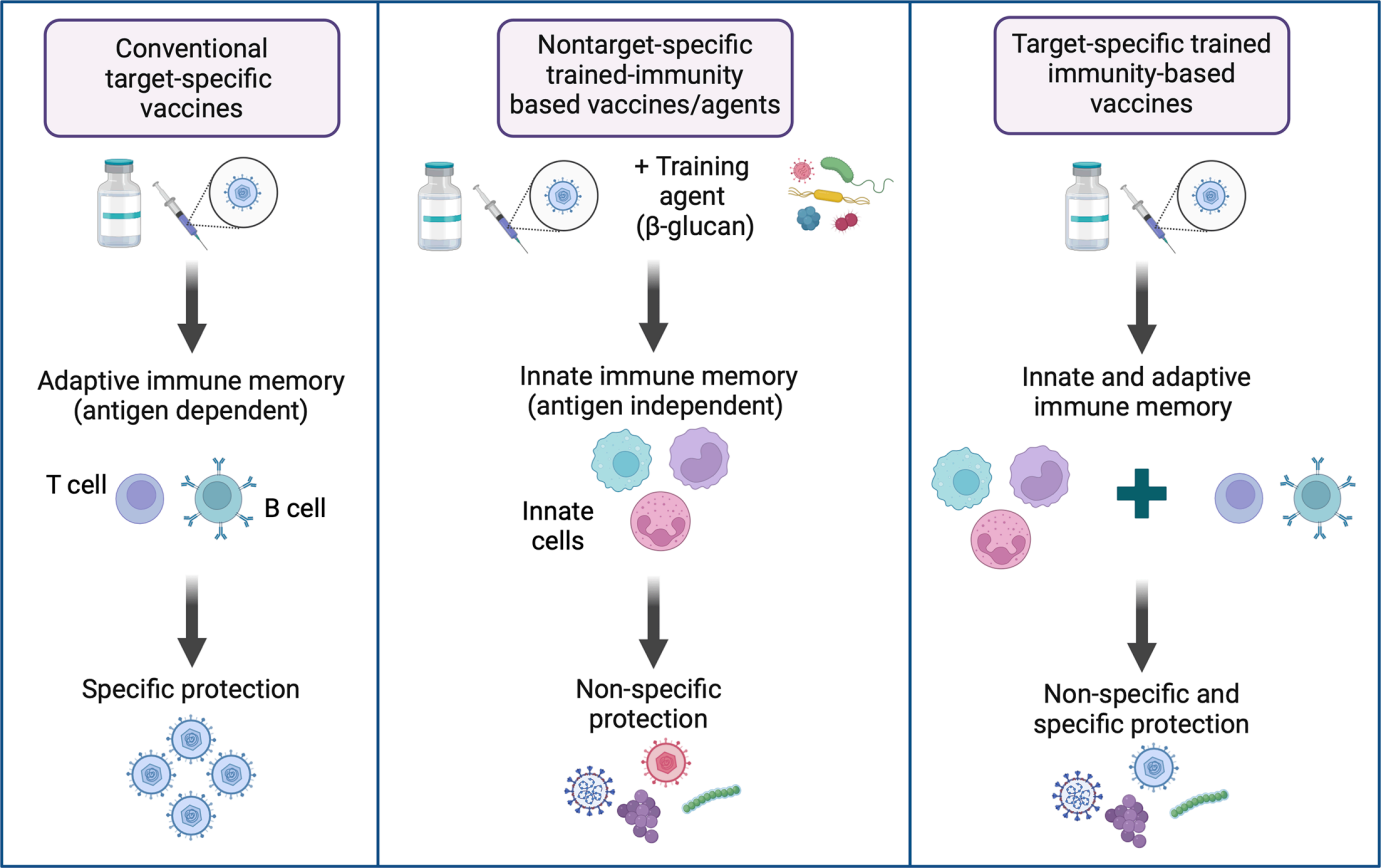
Illustrated paradigms of conventional vaccines, nontarget-specific trained innate immunity (TII)-based vaccines/agents, and next-generation vaccine strategies. Conventional vaccines are primarily designed to target pathogen-specific antigens, leading to antigen-dependent induction of adaptive immune memory and protection. However, some of these vaccines are also capable of a degree of TII. In comparison, nontarget-specific trained immunity-based vaccines or immune agents aim to induce innate immune memory in innate cells and provide rapid, nonspecific (antigen-independent) broad innate immune protection against a variety of heterologous infections. Such strategies can be used for emergent deployment at the onset of pandemics before target-specific vaccines become available. On the other hand, to better control current infectious threats and prepare for future pandemics, the next-generation vaccine strategies should be not only target pathogen-specific but also TII-based, aiming to induce robust and long-lasting innate and adaptive immune memory capable of protection against both the target pathogen and unrelated pathogens via both nonspecific and specific protective mechanisms. One such strategy is respiratory mucosal immunization with a viral-vectored multi-antigenic vaccine for induction of tripartite mucosal immunity consisting of trained innate immunity, mucosal antibody responses, and tissue-resident T cell immunity. Created using BioRender.com.

Harnessing TII offers a promising avenue for development of new intervention strategies against the current and emerging infectious threats where pathogen-specific vaccines may not yet be available. Compared to conventional vaccines that are designed to primarily induce target pathogen-specific adaptive immunity and protection (Figure 2), nontarget-specific TII-based vaccine or agent is designed to train innate immune cells and offer broad nonspecific TII against a variety of pathogens. In this case, a manufactured, scaled-up vaccine with known TII-inducing effects, developed for an unrelated pathogen, could be quickly deployed as a ‘bridge vaccine’ for controlling new emerging infectious threats. Similarly, the biologic agents, particularly PAMPs (pathogen-associated molecular patterns) such as β-glucan, are able to induce centrally induced or tissue-resident TII and thus can also be employed for the same purpose. These could serve as a critical measure to bridge the gap before the target-specific vaccine becomes available (Figure 2).

Conversely, we should also harness our knowledge in TII to continue to develop next-generation vaccine strategies that are both target-specific and TII-based. Such vaccines are expected to induce robust and persisting innate and adaptive immune memory responses, thus offering both nonspecific and specific immune protection (Figure 2). During the COVID-19 pandemic, the nonspecific protective effects of the BCG vaccine were speculated to extend heterologous protection against SARS-CoV-2. However, clinical trials examining whether BCG-induced TII protects against COVID-19 showed contradictory results (Noble et al., 2023). Some trials showed a significant reduction in the development of COVID-19, whereas others showed BCG vaccination to have no effect on the development of COVID-19 (Pittet et al., 2023; Tsilika et al., 2022). Understanding the best ways to exploit off-target effects of the BCG vaccine will be of help to designing next-generation vaccination strategies. Rational design of next-generation vaccine strategies involves the prudent consideration of the route of vaccine delivery, choice of viral vector, and multi-antigenicity. Both preclinical and clinical evidence suggests the deep respiratory mucosal delivery of chimpanzee Ad-vectored multi-antigenic vaccine to effectively elicit tripartite mucosal immunity consisting of TII, mucosal antibody responses, and tissue-resident memory T cell immunity (Afkhami et al., 2022; Jeyanathan et al., 2025). This type of vaccine-induced mucosal immunity is expected to offer the most effective protection against the intended target pathogen and a wide range of unrelated respiratory pathogens.

The emerging knowledge also suggests that different human vaccines and immunization strategies have differential effects on induction of TII (Benn et al., 2023). Specifically, the live and non-live vaccines exhibit differential outcomes of heterologous protection. Live vaccines demonstrate beneficial nonspecific effects and improve overall health and mortality, whereas non-live vaccines have negative nonspecific effects and increase overall all-cause mortality largely in females (Benn et al., 2020). Additional investigation is required to understand such discrepancies between males and females. Furthermore, the current childhood vaccine programs, especially in developing countries, may consider favoring the use of live vaccines over non-live vaccines as the former are more effective in inducing TII. There is also the evidence that the formula of the most recently administered vaccine dictates the presence of residual TII-inducing effects, and that combined delivery of live and non-live vaccines may introduce variable or confounding heterologous protective benefits (Benn et al., 2020).

## Conclusions and future directions

The concept of innate immune memory and TII has advanced significantly over the past decade or so, revealing complex interactions between tissue-resident macrophages and circulating innate immune cells, in response to various immunological exposure. While TII offers promise for enhancing innate immune protection against unrelated pathogens, lung injury, and tumors, it could constitute a mechanism underlying maladaptive inflammation or tissue damage. Overall, it is important to note that induction of innate immune memory and TII following either systemic or local exposure is not created in the same way in terms of innate immune functional outcomes and mechanisms. The differential outcomes of TII in various infection contexts, particularly regarding disease tolerance, highlight the necessity for precise strategies in vaccine and immunotherapy development. As research continues, the coming years are poised to yield deeper insights into the mechanisms underlying TII, potentially revolutionizing clinical approaches to immune modulation and disease prevention.

Moving forward, further investigation is needed to address the longevity of trained tissue-resident macrophages and TII, as currently many in vivo studies were performed during acute phases of the immunological response. We still understand relatively little about the interaction of memory macrophages with tissue structural cells and other immune cells at barrier tissues. We should also make an effort to reach into under-investigated barrier tissue sites, particularly the gut and urogenital tract. More research is required to understand the nature of innate immune environment following multiple immunological exposure. The impact of systemic immunological stimuli on local innate memory and TII at barrier tissue sites needs to be further explored. Key knowledge gaps still remain, and all these questions remain to be addressed at both ends of the life spectrum. Furthermore, we need to continue to study the commonalities and differences and the potential intersection between trained tissue-resident macrophage subsets generated following local Th1 and Th2 immunological exposure.
